# The combination of strip meniscometry and dry eye–related quality-of-life score is useful for dry eye screening during health checkup: Cross-sectional study

**DOI:** 10.1097/MD.0000000000012969

**Published:** 2018-10-26

**Authors:** Sho Ishikawa, Masaru Takeuchi, Naoko Kato

**Affiliations:** a Department of Ophthalmology, Saitama Medical University; b Department of Ophthalmology, National Defense Medical College, Saitama, Japan.

**Keywords:** dry eye, dry eye–related quality-of-life score, strip meniscometry

## Abstract

Strip meniscometry (SM) is a new method for quantification of tear volume using meniscometry strips inserted into the tear meniscus for 5 seconds. The dry eye (DE)-related quality-of-life score (DEQS) questionnaire comprises 15 questions regarding bothersome ocular symptoms and their impact on daily life. These 2 examinations require a relatively short time and are appropriate as screening tests. We evaluated the sensitivity and specificity of SM and DEQS for screening for DE syndrome during general health checkup.

This study included 333 right eyes from 333 soldiers (331 men, 2 women; mean age, 42.8 ± 8.8 years) who underwent health checkups at the Yokosuka Medical Squadron between November and December 2013. We administered the DEQS questionnaire to the subjects. The fluorescein tear film break-up time and fluorescein and rose bengal staining scores were evaluated. A positive outcome was considered when DEQS >15 and SM scores <5 mm. We compared DEQS and SM between a DE group, suspected-DE group and normal group.

Thirty-four (11%) soldiers were diagnosed with definite DE based on the Japanese DE diagnostic criteria. The sensitivities of SM, DEQS, and SM combined with DEQS for definite DE were 71%, 79%, and 59%, respectively, whereas the corresponding specificities were 85%, 91%, and 97%, respectively. None of the enrolled subjects experienced complications such as eye pain or discomfort, except for 1 soldier (0.003%) with conjunctivochalasis, who experienced irritation upon SM.

The results of our study indicate that the combination of SM and DEQS is useful for the detection of DE with high sensitivity and specificity during routine health check-up.

## 1 Introduction

According to the International Dry Eye Workshop, dry eye (DE) syndrome is a multifactorial disease of the tears and ocular surface.^[1]^ It causes various complications, such as ocular discomfort, visual disturbance, tear-film instability, and damage to the integrity of the ocular surface. The prevalence of DE syndrome is high. A study estimated the prevalence of DE syndrome among male and female office workers in Japan to be 60.2% and 76.5%, respectively.^[2]^ DEs lead to general health issues, headache, and vomiting.^[3,4]^ Mental health problems have been reported to be possible risk factors for DE syndrome.^[5]^ Because of the potential for deficits in work performance and the productivity loss associated with these deficits,^[6]^ detection of DE syndrome among employees is important for companies or offices.

Strip meniscometry (SM), which was introduced by Dogru et al,^[7]^ is a new technique for the evaluation of tear-film volume using meniscometry strips designed to absorb the tear meniscus without coming in contact with the conjunctiva. The polyethylene terephthalate strip has a central ditch composed of a urethane-based material, which contains natural blue dye 1. When the strip is applied to the lateral lower-lid tear meniscus for 5 seconds, tears are absorbed forward to the central ditch and are then stained. The SM score is determined based on the length of the stained tear column.

The DE-related quality-of-life score (DEQS) questionnaire is a validated diagnostic tool in Japan.^[8]^ It comprises 15 questions regarding bothersome ocular symptoms and their impact on daily life. The DEQS questionnaire is a reliable tool for evaluating the multifaceted effects of DE syndrome on the daily life of patients, including its effects on mental health. This questionnaire can be easily administered in routine clinical practice. Sakane et al^[8]^ reported an average DEQS of 6.0 in subjects without DE and that of 33.7 in patients with DE. However, no study until now has evaluated the optimal cutoff score for evaluation of DE syndrome. We aimed to evaluate the efficacy, safety, and efficiency of SM, DEQS, and a combination of the 2 tests in the screening of DE syndrome during medical health checkup.

## 2 Methods

The study was approved by the ethical committee of the National Defense Medical College. Informed consent was obtained from each subject. The study adhered to the tenets of the Declaration of Helsinki.

### 2.1 Subjects

We evaluated 333 right eyes from 333 soldiers (mean age, 42.8 ± 8.8 years; 331 men and 2 women) who underwent health checkups at the Yokosuka Medical Squadron between November and December 2013. Subjects who wore contact lenses were excluded. DE was diagnosed according to the Japanese DE diagnostic criteria based on the presence of DE symptomatology; qualitative or quantitative disturbance of tear film [Schirmer value ≤5 mm or fluorescein tear film break-up time (FTBUT) ≤5 s]; and conjunctivocorneal epithelial damage (fluorescein, rose bengal, or lissamine green staining score ≥3 points). Definite DE was diagnosed based on the fulfillment of all 3 criteria. Eyes that fulfilled 2 of the 3 criteria were considered suspicious for DE. ^[1]^ Ophthalmic examination included slit-lamp microscopy and administration of the DEQS questionnaire. FTBUT and fluorescein and rose bengal staining scores were determined after the evaluation of SM scores. We asked the subjects regarding any symptoms, irritation, discomfort, or abnormal touch sensation soon after each test. The data were obtained from health checkups at the Yokosuka Medical Squadron between November and December 2013.

### 2.2 Strip meniscometry

We performed SM using SMTube (Echo Electricity, Fukushima, Japan). The strip was applied to the lateral lower-lid tear meniscus for 5 seconds without touching the ocular surface.

### 2.3 Vital staining

A preservative-free solution (2 μL) containing 1% each of fluorescein and rose bengal dyes was instilled into the conjunctival sac using a micropipette. Fluorescein staining scores and FTBUT were measured first. This was followed by assessment of rose bengal staining scores. Fluorescein and rose bengal staining scores were assigned on a scale of 0 to 9,^[9,10]^ with scores ≥3 considered to be abnormal. FTBUT was measured using fluorescein solution without anesthesia. Subjects were instructed to blink several times to ensure adequate mixing of the fluorescein dye into the tear film. The interval between the last complete blink and appearance of the first corneal black spot in the stained tear film was measured 3 times, and the average value was considered for statistical analysis. FTBUTs <5 seconds were considered abnormal.^[11]^

### 2.4 Dry eye–related quality-of-life score

The DEQS questionnaire, which comprised 15 questions, was administered to the subjects.^[8]^ The frequency and severity of each symptom were scored on a scale of 0 to 4. Frequency was scored as follows: 0, never; 1, occasionally; 2, sometimes; 3, often; or 4, always. Severity was scored as follows: 1, hardly bothered; 2, bothered a little; 3, bothered; and 4, bothered very much. Summary scores were calculated as follows: summary score = (sum of scores for all questions answered) × 25/(total number of questions answered). Summary scores ranged from 0 to 100, with higher scores representing greater disability. In addition, subjects were asked to score their general ocular symptoms and quality of life (QOL) on a scale of 1 to 6, with 1 indicating very good QOL and 6 indicating poor QOL.

### 2.5 Statistical analysis

Statistical analysis was performed using JMP version 10 software (SAS Institute, Tokyo, Japan). Comparisons of SM scores, FTBUTs, staining scores (fluorescein and rose bengal), and DEQS were performed using the Mann-Whitney *U* test. Correlations of SM scores with FTBUTs, staining scores, and DEQS were evaluated using Spearman correlation coefficient analysis. Values of *P* < .05 were considered statistically significant. *P* values <.001 are reported as *P* < .001.

## 3 Results

Of the 333 soldiers enrolled in this study, 34 (11%) male soldiers were diagnosed with definite DE and 28 (8%) male soldiers were suspected of having DE syndrome. Of the 28 eyes with suspected DE, 25 exhibited DE symptoms and short FTBUTs with no conjunctivocorneal epithelial damage, whereas the remaining 3 eyes exhibited disturbance of tear film and conjunctivocorneal epithelial damage, but no DE symptoms. In the definite DE group, 1 soldier (2.8%) had been previously diagnosed with DE syndrome, whereas 10 (28.6%) had used over-the-counter eye drops without consulting an ophthalmologist. Of the 37 soldiers who had received laser in-situ keratomileusis, 5 (14.7%) were diagnosed with definite DE (Table 1).

**Table 1:**
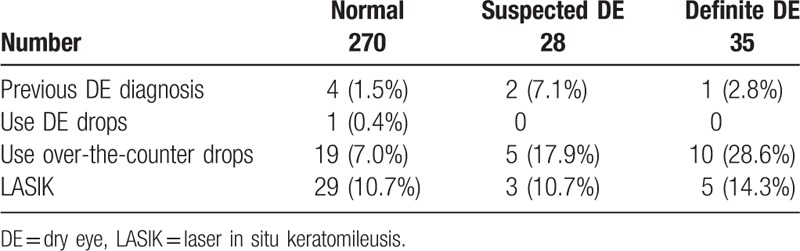
Background of enrolled patients and incidence dry eye.

The SM scores of the subjects in definite DE group (4.8 ± 1.6) were significantly lower than those of the subjects in the normal group (6.4 ± 2.0, *P* < .001). However, the SM scores of the subjects in the suspected DE group (5.8 ± 1.7) were not significantly different from those of the subjects in the normal group. None of the soldiers reported discomfort after SM, except for 1 soldier (0.003%) with conjunctivochalasis, who experienced irritation following SM. In contrast, 72 soldiers (21.6%) complained of irritation, foreign-body sensation, and eye pain following rose bengal dye staining. The mean FTBUT of the definite DE group (4.6 ± 0.7 seconds) was significantly shorter than those of the normal and suspected DE groups (9.3 ± 1.6 and 3.8 ± 0.9 seconds, respectively; both *P* values <.001). The mean FTBUT of the suspected DE group (3.8 ± 0.9) was shorter than that of the definite DE group (4.7 ± 1.7, *P* < .001). Staining scores of the definite DE group (3.0 ± 0.6) were significantly higher than those of the normal and suspected DE groups (0.4 ± 1.0 and 0.5 ± 0.9, respectively; *P* < .001).

**Figure 1:**
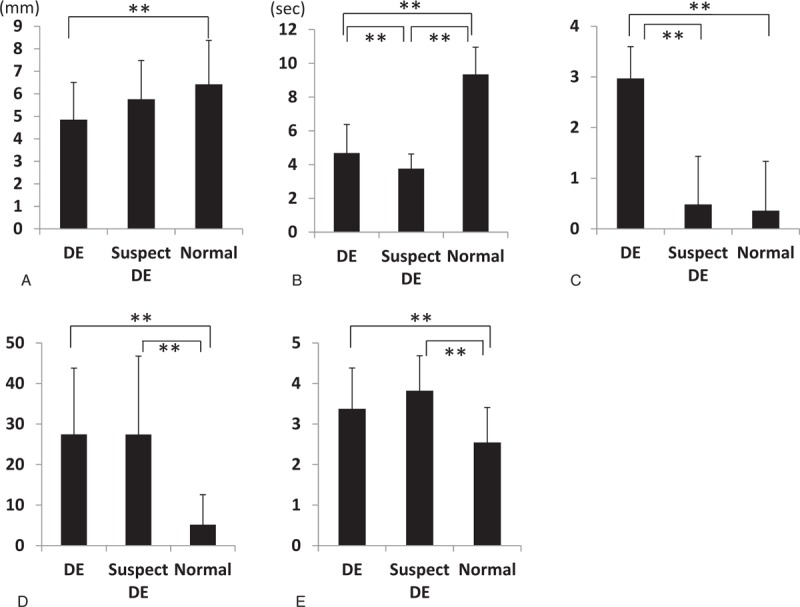
Results of the DE screening test. A, Strip meniscometry scores in the DE group were significantly lower than those in the normal group (*P* < .001), but not significantly different from those in the DE-suspected group (*P* = .061). B, FTBUTs in the DE group were significantly shorter than those in the normal and DE-suspected groups (both *P* values < .001). FTBUTs in the DE-suspected group were significantly shorter than those in the normal group (*P* < .001). C, Staining scores in the DE group were significantly higher than those in the normal and DE-suspected groups (both *P* values < .001). D, Dry eye–related quality-of-life scores (DEQSs) in the DE group were significantly higher than those in the normal group (*P* < .001), but not significantly different from those in the DE-suspected group (*P* = .980). The DE-suspected group exhibited higher DEQSs than the normal group (*P* < .001). E, Quality-of-life (QOL) scores in the DE group were significantly lower than those in the normal group (*P* < .001), but were not different from those in the DE-suspected group (*P* = .051). The DE-suspected group exhibited lower QOL scores than the normal group (*P* < .001). ^∗∗^*P* < .01. DE = dry eye.

The mean DEQS of the definite DE group (27.5 ± 16.3) was significantly higher than that of the normal group (5.2 ± 7.4, *P* < .001), but not significantly different from that of the suspected DE group (27.4 ± 19.4, *P* = .980). The suspected DE group exhibited significantly higher DEQS than the normal group (*P* < .001). The mean QOL scores of the definite (3.4 ± 1.0) and suspected (3.8 ± 0.9) DE groups were both significantly worse than that of the normal group (2.5 ± 0.9, both *P* values < .001, Fig. 1).

**Figure 2:**
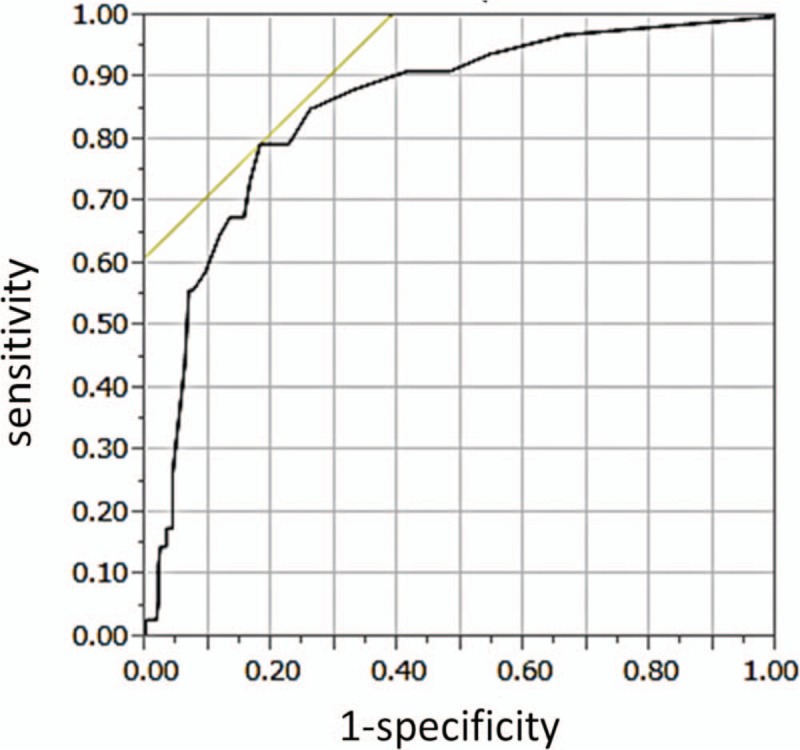
Receiver operating characteristic (ROC) curves for sensitivity and specificity of dry eye–related quality-of-life score (DEQS). The area under the curve calculated based on the ROC was 0.904. The optimal cutoff value for DEQS was >15, which yielded a sensitivity of 79.4% and a specificity of 90.6%.

**Figure 3:**
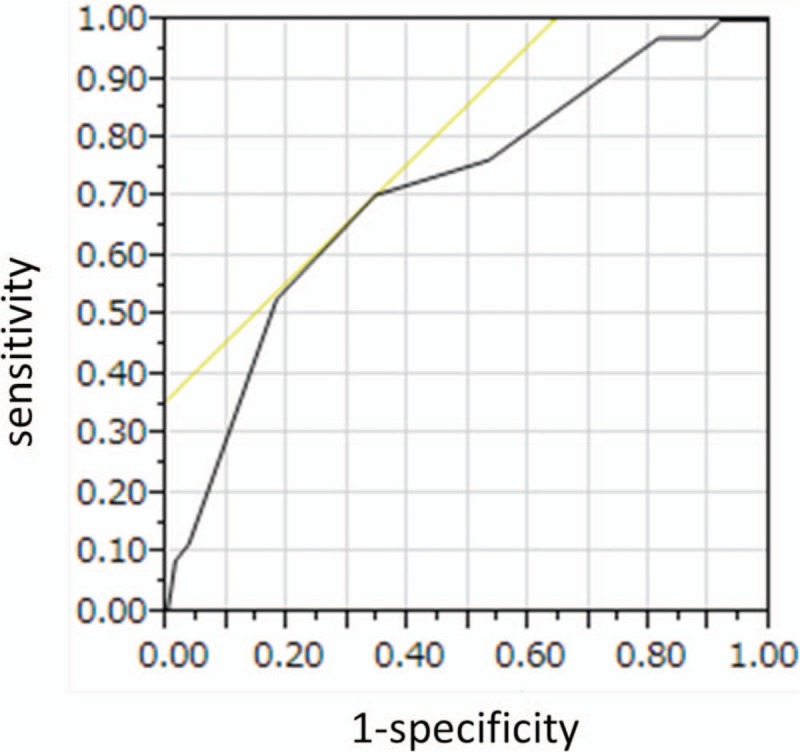
Receiver operating characteristic (ROC) curve for sensitivity and specificity of strip meniscometry (SM). The area under the curve calculated based on the ROC was 0.723. The optimal cutoff value for SM was ≤5 mm, which yielded a sensitivity of 70.6% and specificity of 84.6%.

In order to determine the sensitivities and specificities of SM, DEQS, and the combination of the 2 for the detection of DE, we calculated the area under the curve (AUC) for both SM and DEQS using their respective receiver operating characteristic curves. When we include suspect-DE group in normal group, the AUC for the SM values was 0.723 (Fig. 2, Table 2), and the optimal cutoff value for SM was ≤5 mm, which yielded a sensitivity of 70.6% and specificity of 84.6%. The AUC for the DEQS was 0.904 (Fig. 3, Table 3), and the optimal cutoff value for DEQS was >15, which yielded a sensitivity of 79.4% and specificity of 90.6%. We evaluated the sensitivity and specificity of the combination of SM and DEQS for the diagnosis of DE. The sensitivity and specificity of SM combined with DEQS while considering a single-positive outcome were 97% and 77%, respectively. In contrast, when considering a double-positive outcome for SM in combination with DEQS, the sensitivity increased to 97.3%, whereas the specificity decreased to 58.8% (Table 4).

**Table 2:**
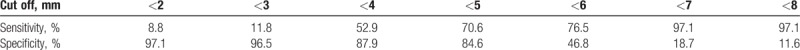
Cut off score, sensitivity, and specificity for strip meniscometry.

**Table 3:**
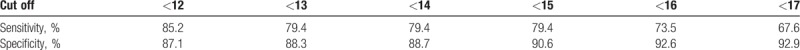
Cut off score, sensitivity, and specificity for dry eye–related quality-of-life score.

**Table 4:**
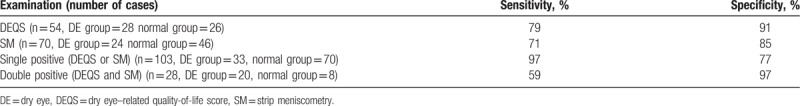
Sensitivity and specificity of dry eye–related quality-of-life score, strip meniscometry score.

## 4 Discussion

Here we evaluated the efficacy and safety of SM and DEQS for the screening of DE syndrome during medical checkup. The SM scores in the definite DE group were significantly lower than those in the normal and suspected DE groups, whereas the DEQSs in the definite DE group were higher than those in the normal group. Both tests exhibited good sensitivity and specificity. The combination of SM and DEQS exhibited high sensitivity when considering a single-positive outcome and high specificity when considering a double-positive outcome.

SM has 4 major advantages for the screening of DE. First, measurement of tear volume by SM requires only 5 seconds. In contrast, Schirmer test, which is the most widely used method for evaluation of tear secretion, requires 5 minutes. Despite this difference, SM findings are positively correlated with those obtained using the Schirmer-1 test.^[7]^ Therefore, we believe that SM is appropriate for the screening of large populations. Second, SM only requires a small volume of tears for measurement of tear volume. A single SM test with only 1 μm of tears produces results with acceptable accuracy^[7]^ and allows for further ophthalmic examinations for DE shortly after SM. Third, the SM strip does not touch the ocular surface or eyelids during examination, and therefore causes relatively less eye pain. In fact, in the present study, only 1 soldier (0.003%) with conjunctivochalasis experienced irritation upon SM. Finally, SM is inexpensive and saves space. Recently developed methods for the screening of DE syndrome, such as tear meniscus height measurement by optical coherence tomography, video-guided tear film break-up time (BUT) measurement,^[12]^ and measurement of biomarkers in tears,^[13]^ require large and expensive devices, complicated techniques, and substantial time. These techniques are thus unsuitable for DE screening during medical checkup. Tear osmolarity evaluation^[14]^ is also a rapid (around 10–15 seconds) test. However, it does require special devices. Furthermore, tear osmolarity evaluation is not included in Japanese DE criteria. We therefore did not use tear osmolarity in this study. Because of its several advantages, SM can help resolve these issues.

In the present study, we conducted medical interviews using the DEQS questionnaire. The DEQS questionnaire has been reported to correlate well with the mental component of the Short Form-8 and the 4 subscales (ocular pain, near vision, distance vision, and mental health) of the National Eye Institute visual function questionnaire-25.^[8]^ When performing screens within companies or offices, it is important to determine the degree of severity of disease that affects work performance and productivity. In Japan, the DEQS is used for DE screening and for the evaluation of the efficacy of DE treatment. However, the cutoff point for DE diagnosis by DEQS has not been evaluated in detail. The results of the present study indicate that the cutoff score for DE diagnosis by DEQS may be set at 15. Sakane et al^[8]^ reported an average DEQS of 33.7 in patients with DE and that of 6.0 in subjects without DE. This indicates consistency with the cutoff value established in the present study.

Combinations of diagnostic tests may help to diagnose DE syndrome more accurately.^[15]^ Some DE patients may have had no symptoms regardless of the objective findings of DE and some may have had severe symptoms without DE findings. However, our evaluations of single- and double-positive outcomes using the combination of SM and DEQS resulted in very high sensitivity and specificity values, respectively. In order to selectively emphasize sensitivity or specificity, we can consider either single- or double-positive outcomes when using the combination method.

A possible limitation or bias in the present study was that we performed medical check-up among soldiers, most of whom were healthy male sailors usually living in the same environment. However, the observed incidence of DE in the present study was 11%, which is consistent with the previously reported incidence of DE in the male population in Japan (8%–12.5%).^[2]^ This suggests that the present results reflect the efficacy of the DE test during medical check-up in the general public.

In conclusion, the present study demonstrated the efficacy, safety, and efficiency of SM and DEQS for the screening of DE syndrome during general medical checkup in a large population. The combination of SM and DEQS for diagnosis of DE yielded high sensitivity and specificity, indicating its feasibility for the screening of DE syndrome.

## Author contributions

**Conceptualization:** Sho Ishikawa.

**Data curation:** Sho Ishikawa.

**Formal analysis:** Sho Ishikawa.

**Investigation:** Sho Ishikawa.

**Methodology:** Sho Ishikawa, Naoko Kato.

**Project administration:** Sho Ishikawa.

**Resources:** Sho Ishikawa.

**Software:** Sho Ishikawa.

**Supervision:** Masaru Takeuchi, Naoko Kato.

**Validation:** Masaru Takeuchi, Naoko Kato.

**Writing – original draft:** Sho Ishikawa.

**Writing – review and editing:** Masaru Takeuchi, Naoko Kato.

Sho Ishikawa orcid: 0000-0002-8729-7286.
